# Bio-hybrid inorganic microparticles derived from CO_2_ for highly efficient and selective removal of antibiotics

**DOI:** 10.1186/s13036-018-0113-8

**Published:** 2018-09-06

**Authors:** Sang Hoon Kim, Ee Taek Hwang, Man Bock Gu

**Affiliations:** 1 Department of Biotechnology, College of Life Sciences and Biotechnology, Korea University, Anam-dong, Seongbuk-gu, Seoul, 136-701 Republic of Korea; 2 Center for Convergence Bioceramic Materials, Korea Institute of Ceramic Engineering & Technology, Cheongju-Si, Chungcheongbuk-do 28160 Republic of Korea

**Keywords:** Aptamer, Antibiotics, Bio-hybrid inorganic microparticle, Selective chemical capturing, Calcium carbonate

## Abstract

**Background:**

Antibiotics, which are the most important medication in human history, have brought global concerns due to their potential risk to human health and environment by accelerating the development of drug-resistant bacteria, and accumulating in the food chain system. Among antibiotics, oxytetracycline (OTC) is widely used in aquaculture, and its potential risk of toxicity to human by bioaccumulation has been reported. Therefore, the effective removal of OTC is highly needed.

**Results:**

In this study, we report bio-hybrid inorganic microparticles (apt-mag-SiCC) for efficient capturing and facile magnet-based separation of oxytetracycline (OTC). These bio-hybrid inorganic microparticles are composed of magnetic separable silica coated calcium carbonate microparticles (mag-SiCC) derived from CO_2_, conjugated with oxytetracycline binding aptamers (OBA). These bio-hybrid inorganic microparticles were successfully synthesized, based on the characterization data obtained by SEM, FT-IR, EDAX, BET, and CLSM. About 6 μm sized bio-hybrid inorganic microparticles showed low non-specific adsorption to OTC and other molecules, and the selective capturing towards to the OTC in both buffer and tap water. Moreover, these bio-hybrid mineral microparticles were found to be stable, even after the repeated usages, maintaining the initial capturing efficiency.

**Conclusion:**

Using the newly synthesized bio-hybrid inorganic microparticles, we could successfully capture OTC by facile magnet-based separation. With advantages of theses bio-hybrid inorganic microparticles such as easy fabrication, low-price, and environmental friendliness, this novel material could be utilized in the drinking water treatment, in vitro medicinal diagnostics, or in vitro removal of antibiotics lining out from the blood (blood purification).

**Electronic supplementary material:**

The online version of this article (10.1186/s13036-018-0113-8) contains supplementary material, which is available to authorized users.

## Background

Antibiotics, which are the most important medication in human history, have brought global concerns due to their potential risk to human health and environment [1, 2]. Up to now, more than 250 antibiotics has been registered, and it is excessively used not only in human medicinal treatment but also in the livestock industry and aquacultures [3]. They can contaminate the soils, ground waters, drinking waters through the various routes [4]. Eventually, it accelerates the development of drug-resistant bacteria, and its residues can be bio-concentrated through the food chain [5]. Among antibiotics, oxytetracycline (OTC) is widely used for shrimp farming, and it is known to be very toxic to the algae which form the basis of the food web [6]. Also, it has been asserted that OTC can be transferred to human by bioaccumulation in the aquatic system [7]. Therefore, there are growing attention to the exposure of OTC, and its effective removal is highly needed.

There are diverse methods for removing and degrading antibiotics, such as oxidation and adsorption techniques [8, 9]. However, such techniques possess disadvantages including intensive energy consumption, toxicity concerns after treatment, difficulty in repetitive usages and so on [10, 11]. Thus, specific capturing of antibiotics using affinity elements such as an antibody, aptamer, and so on, can be one solution to overcome these drawbacks.

Aptamers are well-known affinity receptors for capturing or sensing of small molecules due to their high specificity, affinity, low-cost and reusability [12–18]. The previous report on small organic compounds capturing by aptamers-in-liposomes approach shows high capability in selective capture and elimination of small organic compounds in water [19]. However, this proposed technique is less applicable due to the use of expensive material with poor mechanical stability, and low recovery. In addition, there have been few studies on the efficient capturing property via the aptamer immobilized materials in a solution phase [20, 21].

The calcium carbonate (CaCO_3_) is one of the major biomineral components for structural support in nature. Precise CaCO_3_ architectures can be found in seashells, exoskeletons of algae and spicules of sea urchins [22, 23]. Preparing CaCO_3_ particle is a low-cost and environmentally friendly process because it consumes the carbon dioxide and its morphologies could differ by adding diverse polymer additives to control the crystal nucleation and growth simultaneously [24–29]. Moreover, the characteristics of CaCO_3_, including high mechanical property, thermal stability, and controlled size, promote the efficient use of CaCO_3_ as a mineral material for the bimolecular carrier [30]. However, the fact, that little functional groups on CaCO_3_ results in the poor binding ability and rapid loss of biomolecules from the surfaces [31], limit the extensive use of the calcium carbonate.

Silica offers wide possibilities for surface modification by coupling amines, thiols, carboxyls, and methacrylate groups with specific ligands conjugation [32]. It provides not only the protecting the biomolecules from the loss of viability or reactivity, but also blocking the leakage of the biomolecules from the support material. Moreover, its interconnected pores are likely to give more rapid transport of small molecules, which often enhances the reactivity [33, 34].

Here, by implementing calcium carbonate, silica, and aptamer, we report on a practical and efficient bio-hybrid inorganic microparticles (apt-mag-SiCC) for selective chemical capturing via simple magnetic separation process. These bio-hybrid inorganic microparticles (apt-mag-SiCC) consisted of magnetic separable silica coated calcium carbonate microparticles (mag-SiCC) that are functionalized with oxytetracycline binding aptamers (OBA). In addition to the synthesis and characterization of these bio-hybrid inorganic microparticles, the capturing efficiency of OTC in the buffer as well as tap water were determined. To the best of our knowledge, this is the first study showing the highly efficient capturing of antibiotics using the bio-hybrid inorganic microparticles derived from CO_2_.

## Methods

### Materials

Calcium chloride anhydrate, poly-acrylic acid (PAA), (3-Aminopropyl) trieyhoxysilane (APTES), tetraethylorthosilicate (TEOS), glutaraldehyde solution (GA), ethanolamine, oxytetracycline (OTC), tris(hydroxymethyl)aminomethane, Hexane, FeCl_2_, and FeCl_3_ were purchased from Sigma-Aldrich and used without any additional purification. Oxytetracycline binding aptamer(OBA) was synthesized from GenoTech Corp. (Daejeon, Korea). The sequence was 5’-CGTACGGAATTCGCTAGCACGTTGACGCTGGTGCCCGGTTG TGGTGCGAGTGTTGTGTGGATCCGAGCTCCACGTG -3′. The dissociation constant (*K*_d_) of the OBA was 12.08 ± 2.25 nM [35]. For confocal microscopy, Fluorescence tag (FAM) was modified at 3′ end of the aptamer.

### Preparation and characterization of magnetically separable inorganic microparticles

The magnetic nanoparticles were fabricated by the co-precipitation method by adding a 5 M NaOH solution into a mixed solution of 0.25 M ferrous chloride and 0.5 M ferric chloride (molar ratio 1:2) at pH 11, 90 °C. These particles were washed with distilled water for serval times, then separated from the supernatant and stored in distilled water for further uses. The magnetically separable calcium carbonate microparticles (mag-CC) were synthesized via a simple procedure with some modifications. Briefly, 18 mL of 20 mM CaCl_2_ was dissolved in distilled water and mixed with 400 μL Tris-HCl (1 M, pH 8.8). The solution was sonicated for 60 min at 25 °C and titrated with 0.1 M HCl to pH 8.3. PAA (0.1 mg/ml) and 100 μL of magnetic nanoparticles were dispersed in solution, and distilled water was added to make a total volume of 20 ml. The solution mixture was stirred in a chamber under constant and controlled CO_2_ pressure (10% CO_2_, 30 °C, Thermo Forma Series II water jacketed CO_2_ incubator) at 830 rpm for overnight. After, the fabricated mag-CC were washed with distilled water for five times and stored at 4 °C in distilled water for further uses. The mag-CC were coated with silica by the modified sol-gel process using APTES and TEOS to obtain the amine moiety. Then they were mixed with n-hexane and stirred for 2 min. After adding APTES, the solution was stirred for another 5 min, and TEOS was added to the solution and reacted for 2 h. The volume ratio of mag-CC: n-hexane: APTES: TEOS was 5:10:0.16:0.12. The silica-coated mag-CC (mag-SiCC) were washed five times with distilled water to remove the residues that were not reacted. The mag-SiCC were stored in the distilled water at 4 °C before further use for aptamer conjugation.

The morphological and elemental analyses of the mag-SiCC and mag-CC were investigated by SEM (SEM; SU-70, Hitachi Co. Ltd., Tokyo, Japan) equipped with energy-dispersive X-ray spectroscopy (X-Max^N^ 50mm^2^, Horiba Ltd., Kyoto, Japan). The SEM was used to get the high-resolution image of mag-SiCC and mag-CC. As a sample preparation, each material was dropped on a silicon wafer, and Pt was sputtered for SEM analysis. The elemental analysis of the mag-SiCC and mag-CC were done by energy-dispersive X-ray spectroscopy (EDAX). FT-IR spectra of the mag-SiCC & mag-CC were measured by an FT-IR spectrometer (Agilent Cary 630 FTIR, Agilent Technologies Inc., USA) range of 650–4000 cm^− 1^. XRD patterns were analyzed with a Dmax2500, and Intensity data were collected over a 2θ range of 5° to 80° with 0.02° step size. Pore size and BET (Brunauer-Emmett-Teller) surface were analyzed using a micrometrics ASAP 2420. The pore size was calculated using Barett-Joyner-Halenda method (BJH).

### Preparation of bio-hybrid inorganic microparticles

To prepare bio-hybrid inorganic microparticles for the specific chemical capturing, the aptamers were conjugated to the mag-SiCC by forming covalent bonds between amine-modified aptamers and the mag-SiCC. Briefly, mag-SiCC were dispersed in 1xPBS pH 8.0, and glutaraldehyde (GA) was added to make a final concentration of 0.1%. The solution was incubated for 2 h at room temperature with mild rotating. The activated mag-SiCC solution was washed ten times with distilled water to remove unreacted glutaraldehyde residues and dispersed in 1xPBS pH 7.6. The aldehyde-modified mag-SiCC were mixed with 50 μl of 10 μM amine modified oxytetracycline-binding aptamer (OBA) for OTC capturing. Then, the supernatant was removed by magnetic separation, and the material was washed with PBS buffer for three times. The remaining reactive group on mag-SiCC was blocked by 50 mM of ethanolamine pH 8.0 for 1 h by rotating at room temperature. After washing for three times, materials were kept at 4 °C until further uses.

A confocal laser scanning microscope (CLSM; LSM 700, Carl Zeiss Co. Ltd., Germany) was used to examine the aptamer-conjugated mag-SiCC by fluorescence microscopy. To excite the FAM-labeled aptamer on mag-SiCC, a 488 nm UV laser was used.

### Analysis of elimination of small organic molecules using bio-hybrid inorganic microparticles

To determine the capturing efficiency of bio-hybrid inorganic microparticles (apt-mag-SiCC), 100 μL of prepared materials were incubated with each of 5 μM OTC, Naproxen, Diclofenac, respectively, in a binding buffer solution (100 mM NaCl, 20 mM Tris-HCl, 2 mM MgCl_2_, 5 mM KCl, 1 mM CaCl_2_, pH 7.6) for 2 h with mild shaking at room temperature. This incubation time can be reduced if more concentrated aptamer and mag-SiCC are used. Then, apt-mag-SiCC were magnetically separated from the supernatant, and the unbound OTC supernatant was measured at 368 nm wavelength by UV-Vis spectrophotometer. To determine the capturing efficiency of apt-mag-SiCC in tap water, 100 μl of apt-mag-SiCC were incubated with 5 μM of OTC-spiked tap water for 2 h with mild shaking at room temperature. Unbound OTC was measured with UV-Vis spectrophotometer. For the repetition usage, the apt-mag-SiCC was washed with 1 ml of deionized water for three times and stored in 1 ml of deionized water at 4 °C for overnight. Before using the apt-mag-SiCC, it was washed with 1 ml of binding buffer solution for 3 times. The same chemical capturing protocol was followed for assessing the reusability of the apt-mag-SiCC.

## Results and discussion

### Physico-chemical characteristics of magnetically separable inorganic microparticles

**Fig. 1:**
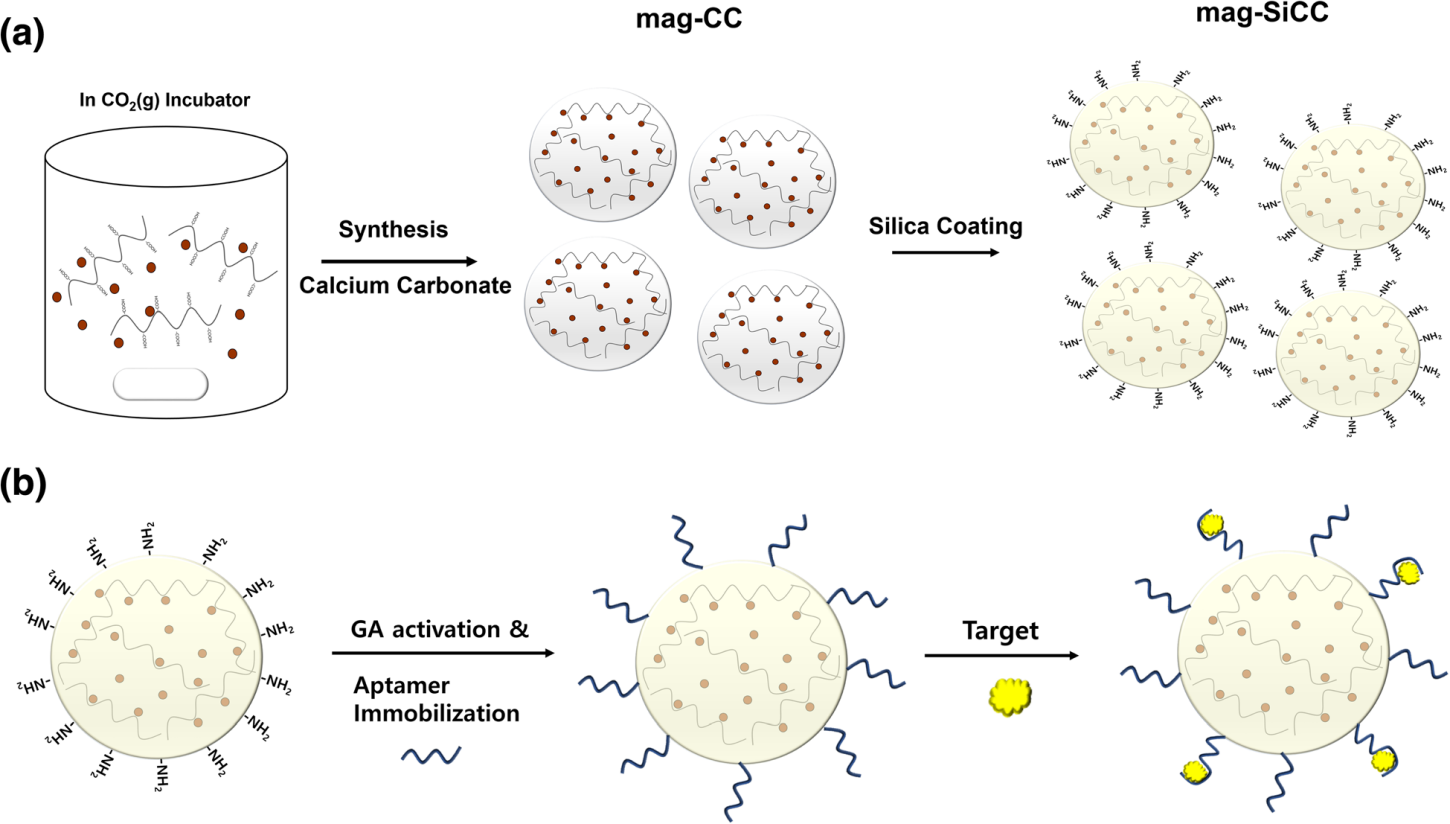
**a** Schematic illustration of the preparation of mag-SiCC. The mag-CC was produced using CO_2_ gas; then it was subjected to silica coating process to provide amine functional group on mag-SiCC for aptamer conjugation. **b** Schematic illustration of preparing the bio-hybrid inorganic microparticles. The aptamers were conjugated to the mag-SiCC for specific capturing of oxytetracycline

**Fig. 2:**
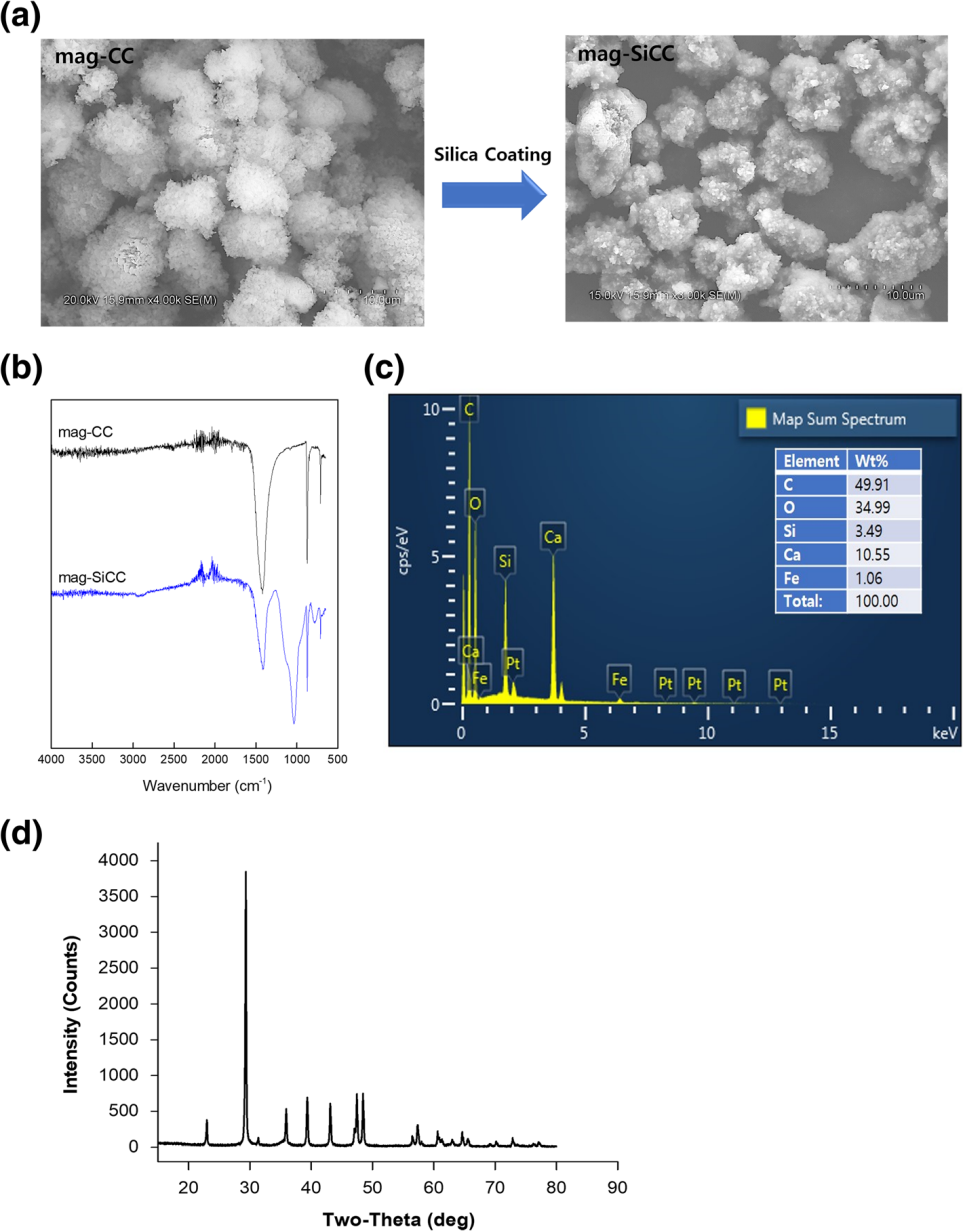
**a** The SEM images of magnetically separable calcium carbonate particles before (mag-CC) and after silica coating (mag-SiCC). **b** FT-IR spectrum of magnetically separable calcium carbonate particles before and after silica coating. **c** Energy-dispersive X-ray spectroscopy (EDAX) of mag-SiCC. **d** XRD patterns of mag-SiCC

The silica-coated magnetically separable calcium carbonate microparticles (mag-SiCC) were successfully synthesized and modulated by adding the polyacrylic acid polymers. The crystallization process of the magnetically separable calcium carbonate microparticles (mag-CC) was performed in the presence of polyacrylic acid polymer and magnetic nanoparticles under constant CO_2_ pressure. Subsequently, silica coating was conducted by adding APTES and TEOS to make the mag-SiCC, depicted in Fig. 1a. As can be seen from Fig. 2a, the anticipation of PAA chains served as repeating negative charge templates for Ca^2+^ ions to attach and grow; thus, the final particles included not a single chain but several chains so that their size increased to micro-sized CaCO_3_ particles. Interestingly, we could observe two different morphologies (pyramid shape and rectangular shape) are present on the CaCO_3_ particles. Furthermore, the sol-gel silica coating of this mag-CC from the precursor of silicon dioxide can carry amine groups with strengthened silica layers. Especially, the use of excess APTES reinforces the overall mechanical strength of inorganic microparticles. Fig. 2a. shows the SEM image of the mag-SiCC particles, which possess amine functional groups, within the micro-size range about 6 μm diameter. This successful silica coating is supported by FT-IR spectrum analysis for with and without silica coated mag-CC (Fig. 2b). From the FT-IR results, we could see the typical CaCO_3_ peak around 1401, 873 and 712 cm^− 1^ for both samples [36]. Only in the silica-coated sample, the Si-O-Si stretching vibration peak was observed at 1068 cm^− 1^ wavenumber, which shows the stable coating of silica on mag-CC surfaces [37]. Additionally, Energy-dispersive X-ray Spectroscopy (EDAX) was used to confirm coating of silica to the mag-CC surface (Fig. 2c). As we expected, Si peak was observed which indicates successful silica-coating of mag-CC from the modified sol-gel process. Furthermore, Fig. 2d shows XRD patterns of the mag-SiCC with peaks at 2θ of 29.34°, 35.96°, 39.34°, and 43.1° which indicates the crystalline phase of the mag-SiCC was calcite [38]. Also, mag-SiCC can be separated from the solution by a magnet because of the presence of magnetic nanoparticles inside the mag-SiCC. As shown in Additional file 1: Figure S1, the mag-SiCC was separated by the magnet within 3 min, and this inorganic microparticle could be used in diverse applications including in vitro blood purification and medicinal diagnostics. The synthesized mag-SiCC revealed a porous morphology by exhibiting the pore-size distribution obtained from the representative N_2_ adsorption and desorption data using the BJH method. The size and volume of these pores in the mag-SiCC are described in Additional file 2: Table S1; the BET surface area, single point total pore volume, average pore size from adsorption, and average pore size from desorption of mag-SiCC were 37.009 m^2^/g, 0.164 cm^3^/g, 19.356 nm, and 16.513 nm, respectively. These unique structures of mag-SiCC enable favorable space for the interaction between the aptamer and the target molecule.

### Selective capture of oxytetracycline using bio-hybrid inorganic microparticles

**Fig. 3:**
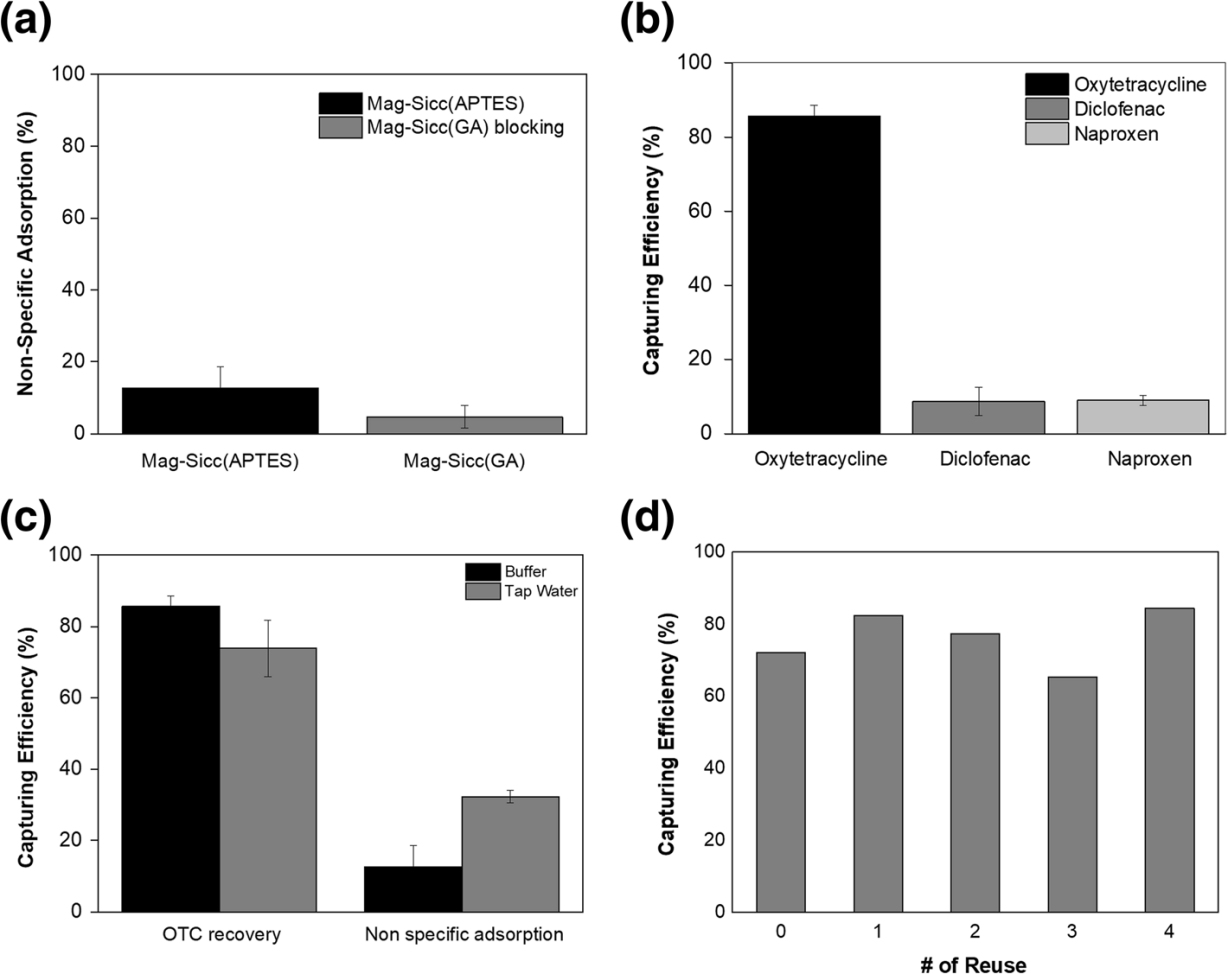
**a** Non-specific adsorption of OTC to mag-SiCC and ethanolamine blocked mag-SiCC after GA activation. **b** Specific capturing of oxytetracycline using bio-hybrid inorganic microparticles (apt-mag-SiCC). OBA aptamer was used to capture OTC (Diclofenac and naproxen were used as counter targets). **c** Capturing efficiency of OTC in buffer solution and spiked tap water using apt-mag-SiCC. **d** Repeated usage of apt-mag-SiCC for five times. Capturing efficiency was calculated at each time point

The use of aptamers in the capturing and separation of certain small molecules play a vital role in the elimination of toxic and hazardous chemicals in the environment. Hence, small-molecule compounds such as antibiotics, pesticide, heavy metals, and so on, can be potential targets for the selection and application of aptamers. To demonstrate the efficient and selective capturing of antibiotics from water by using bio-hybrid inorganic microparticles (apt-mag-SiCC), we chose the oxytetracycline as a model target. For selective capturing of OTC, conjugation of amine-labeled oxytetracycline binding aptamer (OBA) to the mag-SiCC was performed as described in Fig. 1b. The bio-hybrid inorganic microparticles (apt-mag-SiCC) were successfully fabricated for the selective capturing of the OTC. In order to confirm the aptamer immobilization on the mag-SiCC, the confocal laser scanning microscope (CLSM) images of apt-mag-SiCC were obtained by using FAM-labeled aptamers. As clearly shown in Additional file 3: Figure S2, the green fluorescence from FAM on aptamers was observed from all over the mag-SiCC, suggesting that the aptamers were conjugated not only on the certain part of the mag-SiCC but the whole surface of them. Also, the number of aptamers that immobilized on the mag-SiCC (18–20 mg) was found to be around 0.3–0.45 nmol by measuring the concentration of unbound aptamers after conjugation process (Additional file 4: Figure S3). Non-specific adsorption of the target was analyzed on this newly developed mag-SiCC before applying to the capturing of oxytetracyclines. The result in Fig. 3a indicates that both mag-SiCC and ethanolamine blocked mag-SiCC after GA activation show low non-specific binding to the OTC, which is less than 15%.

Since these newly developed mag-SiCC microparticles featured low adsorption to OTC, the specific capturing of OTC on the bio-hybrid inorganic microparticles (apt-mag-SiCC) was analyzed. It is clear, from Fig. 3b, that 85 ± 2.8% of OTC in the buffer solution was captured by the apt-mag-SiCC. However, only 8.7 ± 3.7% and 9.0 ± 1.3% of diclofenac and naproxen were found to be non-specifically adsorbed, which suggests the specific capturing of OTC. The use of bio-hybrid inorganic microparticles was further applied to capture and separate OTC in tap water samples. The same capturing procedure used in buffer was repeatedly conducted for OTC-spiked tap water samples. The capturing efficiency of OTC in tap water sample using this apt-mag-SiCC was about 73 ± 7.9%, which was less efficient, compared to buffer condition (Fig. 3c). This reduction in efficiency seems to be mainly due to the different ionic strength between buffer and tap water samples, in which the tap water sample should have much lower ionic strength. However, this reduced capturing efficiency of OTC in the tap water is still considered to be competitive, compared to our previous study where liposome was used for keeping the same ionic strength because the aptamers could be less functional at lower ionic strength [19]. Interestingly, the efficiency using the aptamers-in-liposome is found to be similar to this study. The reason how the aptamers are somewhat functional in this study, where the ionic strength is considered to be lower, may be due to the pores generated from silica-coating steps. The pores spread on silica could change the microenvironment surrounding aptamers, and so the aptamers were functional in part, even though the ionic strength is lower than the buffer [39].

The repeated usage of bio-hybrid inorganic microparticles (apt-mag-SiCC) for the capturing of the OTC was also investigated. Since the presence of MNPs in the apt-mag-SiCC allowed the magnetic separability of this apt-mag-SiCC from the liquid media, a magnet was used to separate the apt-mag-SiCCs in solution at an extremely short separation time. This feature, the magnetic separability, allows the application of bio-hybrid inorganic microparticles in recovery or separation from the mixtures and the cost reduction by the repeated usages of apt-mag-SiCC. Fig. 3d shows the results of the repeated usage of apt-mag-SiCC. The immobilized aptamers were found to be stable, until the fifth use of recycling; they preserved the initial capturing efficiency of 72% for five days within 7% standard deviation. Regarding the reusability of the aptamers, it is well known that the function or binding affinity of the aptamers are varied upon the buffer condition used (because of the ionic strength in the buffer, which affects the binding affinity of the aptamer). Even if the binding affinity of the aptamer is strong, it could be weaken if the diffrent buffer is used. Therefore, we can modulate the function and binding affinity of aptamers by simply changing the buffers [40–43]. While the apt-mag-SiCC was recycled for five times, there was no significant reduction in the capturing efficiency, and the maximum loss of capturing efficiency was 6.9% at fourth use, compared to the initial efficiency. This feature of repeatability, again, could contribute to the reduction in the cost for removing antibiotics from the water.

## Conclusions

In conclusion, we have shown the efficient and selective chemical capturing by using so-called bio-hybrid inorganic microparticles (apt-mag-SiCC) for the first time. These bio-hybrid inorganic microparticles were characterized by XRD, SEM, FT-IR, EDAX, BET, and CLSM. The size of bio-hybrid inorganic microparticles was ranged between 6 μm in diameter. These bio-hybrid inorganic microparticles revealed a low non-specific binding to the oxytetracycline (OTC) which was less than 15%. The capturing efficiency of the target was found to be about 85% and 73% in the buffer solution and the tap water, respectively, with high specificity and facile magnetic separability. Furthermore, these bio-hybrid inorganic microparticles were stable, even after five times of the repeated usage, showing less than 6% of the reduction in its initial recovery yield of 72%. Regarding the advantages of bio-hybrid inorganic microparticles, including the ease of fabrication, low-cost in production, and reduction of CO_2,_ this novel material could be a robust platform for the toxic chemical capturing and the removal. Furthermore, this new study may provide a novel way to design the aptamer-based platforms and have a great potential for in pharmaceutical and environmental fields.

## Additional files

Additional file 1: **Figure S1.** Digital images of magnetic separation of fabricated mag-SiCC. Samples were separated within 3min. (TIF 707 kb)

Additional file 2: **Table S1.** Physical properties of mag-SiCC. (XLSX 10 kb)

Additional file 3: **Figure S2.** The CLSM image of the FAM-labeled aptamers on mag-SiCC. (TIF 514 kb)

Additional file 4: **Figure S3.** (a) The concentration of aptamers immobilized on mag-SiCC. (b) Capturing efficiency of apt-mag-SiCC by increasing the concentration of the OTC. (TIF 94 kb)

## Abbreviations

APTES: (3-Aminopropyl) trieyhoxysilane
apt-mag-SiCC: Aptamer-conjugated magnetic separable silica coated calcium carbonate microparticles
CaCO_3_: Calcium carbonate
GA: Glutaraldehyde solution
mag-CC: Magnetically separable calcium carbonate microparticles
mag-SiCC: Magnetic separable silica coated calcium carbonate microparticles
OBA: Oxytetracycline binding aptamer
OTC: Oxytetracycline
PAA: Poly-acrylic acid
TEOS: Tetraethyl orthosilicate

## References

[1] Boxall ABA, Ericson JF, Brooks BW, Huggett DB (2012). Environmental fate of human pharmaceuticals. Human Pharmaceuticals in the Environment: current and future perspectives.

[2] Chopra I, Howe TGB, Linton AH, Linton KB, Richmond MH, Speller DCE (1981). The tetracyclines: prospects at the beginning of the 1980s. J Antimicrob Chemother.

[3] Kümmerer K (2003). Significance of antibiotics in the environment. J Antimicrob Chemother.

[4] Xu XR, Li XY (2010). Sorption and desorption of antibiotic tetracycline on marine sediments. Chemosphere.

[5] Rodriguez-Mozaz S, Chamorro S, Marti E, Huerta B, Gros M, Sanchez-Melsio A (2015). Occurrence of antibiotics and antibiotic resistance genes in hospital and urban wastewaters and their impact on the receiving river. Water Res.

[6] SGs K H€o, Wahlstrom A, Poungshompoo S, Bengtsson B-E, Kautsky N (2003). Antibiotic use in shrimp farming and implications for environmental impacts and human health. Int J Food Sci Technol.

[7] Boonsaner M, Hawker DW (2013). Evaluation of food chain transfer of the antibiotic oxytetracycline and human risk assessment. Chemosphere.

[8] Huber MM, Canonica S, Park G-Y, von Gunten U (2003). Oxidation of pharmaceuticals during ozonation and advanced oxidation processes. Environ Sci Technol.

[9] Pinkston KE, Sedlak DL (2004). Transformation of aromatic ether- and amine-containing pharmaceuticals during chlorine disinfection. Environ Sci Technol.

[10] Mehrjouei M, Müller S, Möller D (2014). Energy consumption of three different advanced oxidation methods for water treatment: a cost-effectiveness study. J Clean Prod.

[11] Ahmed MB, Zhou JL, Ngo HH, Guo W (2015). Adsorptive removal of antibiotics from water and wastewater: progress and challenges. Sci Total Environ.

[12] Ellington AD, Szostak JW (1990). In vitro selection of RNA molecules that bind specific ligands. Nature.

[13] Tuerk C, Gold L (1990). Systematic evolution of ligands by exponential enrichment: RNA ligands to bacteriophage T4 DNA polymerase. Science.

[14] Kim Y-J, Kim YS, Niazi JH, Gu MB (2009). Electrochemical aptasensor for tetracycline detection. Bioprocess Biosyst Eng.

[15] Kim YS, Kim JH, Kim IA, Lee SJ, Jurng J, Gu MB (2010). A novel colorimetric aptasensor using gold nanoparticle for a highly sensitive and specific detection of oxytetracycline. Biosens Bioelectron.

[16] Kim YS, Gu MB, Gu MB, Kim H-S (2014). Advances in Aptamer Screening and Small molecule Aptasensors. Biosensors based on aptamers and enzymes.

[17] Kwon YS, Ahmad Raston NH, Gu MB (2014). An ultra-sensitive colorimetric detection of tetracyclines using the shortest aptamer with highly enhanced affinity. Chem Commun.

[18] Seo HB, Kwon YS, J-e L, Cullen D, Noh H, Gu MB (2015). A novel reflectance-based aptasensor using gold nanoparticles for the detection of oxytetracycline. Analyst.

[19] Kim YS, Niazi JH, Chae YJ, Ko UR, Gu MB (2011). Aptamers-in-liposomes for selective and multiplexed capture of small organic compounds. Macromol Rapid Commun.

[20] Kim JH, Hwang ET, K-k K, Tatavarty R, Gu MB (2011). Aptamers-on-nanofiber as a novel hybrid capturing moiety. J Mater Chem.

[21] Martin JA, Phillips JA, Parekh P, Sefah K, Tan W (2011). Capturing cancer cells using aptamer-immobilized square capillary channels. Mol BioSyst.

[22] Politi Y, Arad T, Klein E, Weiner S, Addadi L (2004). Sea urchin spine calcite forms via a transient amorphous calcium carbonate phase. Science.

[23] Meldrum FC, Cölfen H (2008). Controlling mineral morphologies and structures in biological and synthetic systems. Chem Rev.

[24] Hwang ET, Gang H, Chung J, Gu MB (2012). Carbonic anhydrase assisted calcium carbonate crystalline composites as a biocatalyst. Green Chem.

[25] Hwang ET, Gang H, Gu MB (2013). CO2 bioconversion using carbonic anhydrase (CA): effects of PEG rigidity on the structure of bio-mineralized crystal composites. J Biotechnol.

[26] Yu SH, Cölfen H, Hartmann J, Antonietti M (2002). Biomimetic crystallization of calcium carbonate spherules withControlled surface structures and sizes by double-HydrophilicBlock copolymers. Adv Funct Mater.

[27] Yu J, Lei M, Cheng B, Zhao X (2004). Facile preparation of calcium carbonate particles with unusual morphologies by precipitation reaction. J Cryst Growth.

[28] Ouhenia S, Chateigner D, Belkhir MA, Guilmeau E, Krauss C (2008). Synthesis of calcium carbonate polymorphs in the presence of polyacrylic acid. J Cryst Growth.

[29] Gebauer D, Cölfen H, Verch A, Antonietti M (2009). The multiple roles of additives in CaCO3Crystallization: a quantitative case study. Adv Mater.

[30] Dmitry V, Volodkin NIL, Gleb B (2004). Sukhorukov. Protein encapsulation via porous CaCO3 microparticles templating. Biomacromolecules.

[31] Li F, Feng Y, Wang Z, Yang L, Zhuo L, Tang B (2010). Direct electrochemistry of horseradish peroxidase immobilized on the layered calcium carbonate-gold nanoparticles inorganic hybrid composite. Biosens Bioelectron.

[32] Dean SL, Stapleton JJ, Keating CD (2010). Organically modified Silicas on metal nanowires. Langmuir.

[33] Mackenzie JD, Bescher EP (2007). Chemical routes in the synthesis of nanomaterials using the sol–gel process. Acc Chem Res.

[34] Walcarius A, Collinson MM (2009). Analytical chemistry with silica sol-gels: traditional routes to new materials for chemical analysis. Annu Rev Anal Chem.

[35] Niazi JH, Lee SJ, Kim YS, Gu MB (2008). ssDNA aptamers that selectively bind oxytetracycline. Bioorg Med Chem.

[36] Rodriguez-Blanco JD, Shaw S, Benning LG (2011). The kinetics and mechanisms of amorphous calcium carbonate (ACC) crystallization to calcite, viavaterite. Nanoscale.

[37] Roy K, Alam MN, Mandal SK, Debnath SC (2015). Silica-coated nano calcium carbonate reinforced polychloroprene rubber nanocomposites: influence of silica coating on cure, mechanical and thermal properties. J Nanostructure Chem.

[38] Xu B, Poduska KM (2014). Linking crystal structure with temperature-sensitive vibrational modes in calcium carbonate minerals. Phys Chem Chem Phys.

[39] Carrasquilla C, Lau PS, Li Y, Brennan JD (2012). Stabilizing structure-switching signaling RNA aptamers by entrapment in sol-gel derived materials for solid-phase assays. J Am Chem Soc.

[40] Baker BR, Lai RY, Wood MS, Doctor EH, Heeger AJ, Plaxco KW (2006). An electronic, aptamer-based small-molecule sensor for the rapid, label-free detection of cocaine in adulterated samples and biological fluids. J Am Chem Soc.

[41] Baldrich E, Arrays A, Khademhosseini A, Suh K-Y, Zourob M (2011). Biological microarrays: methods and protocols.

[42] Li M, Zhou X, Ding W, Guo S, Wu N (2013). Fluorescent aptamer-functionalized graphene oxide biosensor for label-free detection of mercury(II). Biosens Bioelectron.

[43] Smestad J, Maher IIILJ (2013). Ion-dependent conformational switching by a DNA aptamer that induces remyelination in a mouse model of multiple sclerosis. Nucleic Acids Res.

