# Supplementary figures and images for: Bio-hybrid inorganic microparticles derived from CO2 for highly efficient and selective removal of antibiotics
Source: J Biol Eng. 2018 Sep 6;12:16. doi: 10.1186/s13036-018-0113-8 (PMC6127930; doi:10.1186/s13036-018-0113-8)

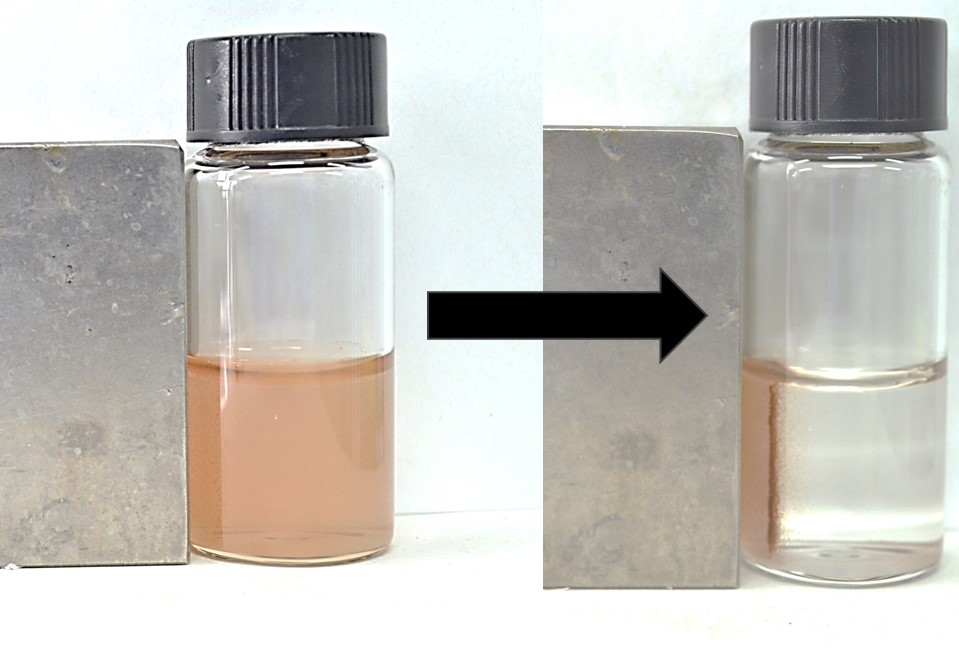

Supplement: Supplementary file 1 — Figure S1. Digital images of magnetic separation of fabricated mag-SiCC. Samples were separated within 3min. (TIF 707 kb) [file 13036_2018_113_MOESM1_ESM.tif]

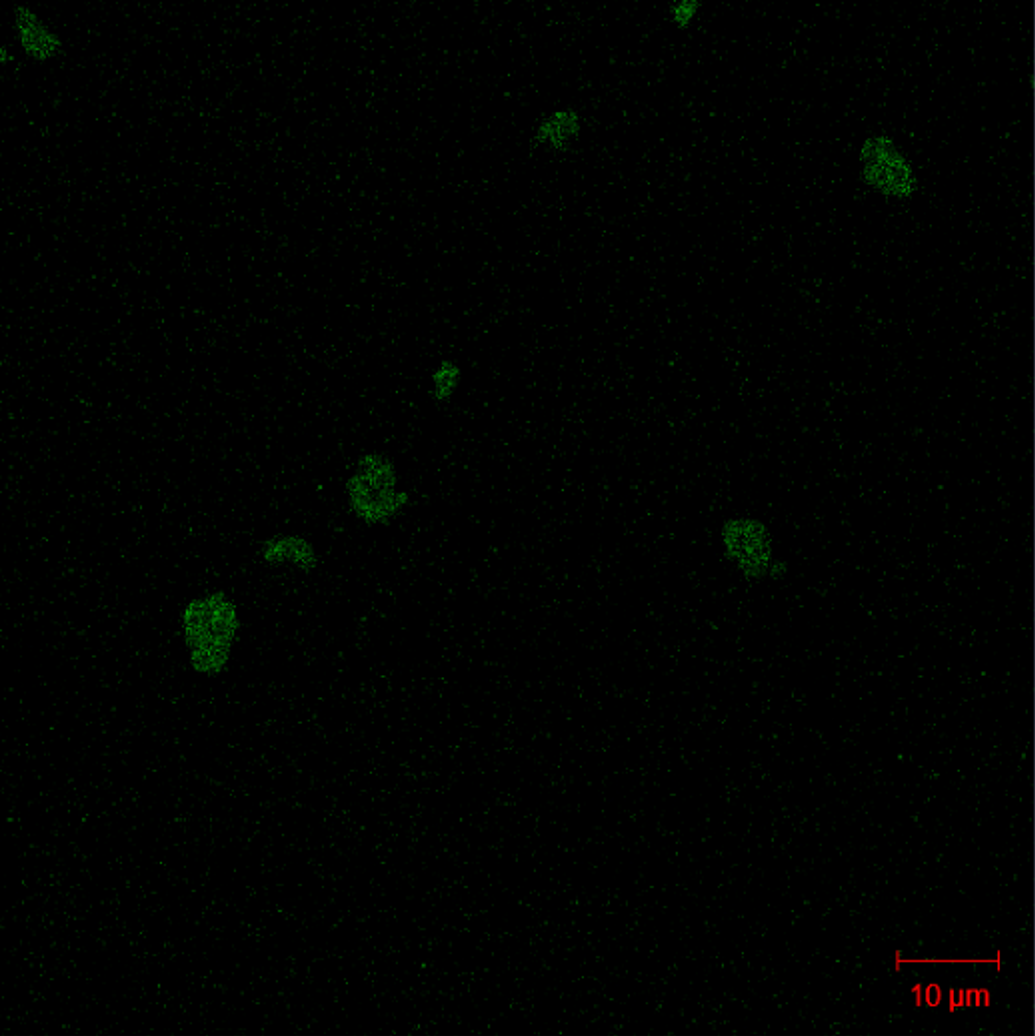

Supplement: Supplementary file 3 — Figure S2. The CLSM image of the FAM-labeled aptamers on mag-SiCC. (TIF 514 kb) [file 13036_2018_113_MOESM3_ESM.tif]

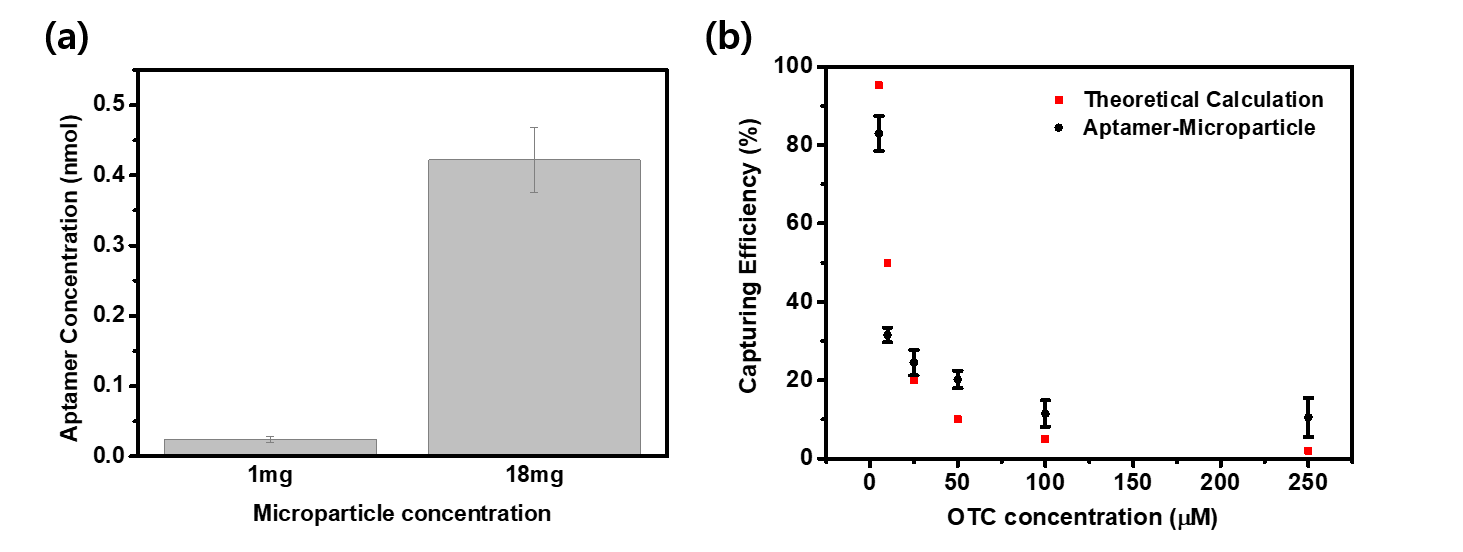

Supplement: Supplementary file 4 — Figure S3. (a) The concentration of aptamers immobilized on mag-SiCC. (b) Capturing efficiency of apt-mag-SiCC by increasing the concentration of the OTC. (TIF 94 kb) [file 13036_2018_113_MOESM4_ESM.tif]
